# CHURCH RELATIONSHIPS, DISCRIMINATION, AND GENERALIZED ANXIETY DISORDER AMONG OLDER AFRICAN AMERICANS

**DOI:** 10.1093/geroni/igz038.2765

**Published:** 2019-11-08

**Authors:** Ann W Nguyen

**Affiliations:** Jack, Joseph and Morton Mandel School of Applied Social Sciences, Case Western Reserve University, Cleveland, Ohio, United States

## Abstract

The African American church has played a major role in African American communities, and church relationships represent an important stress-coping resource for older African Americans. This study examined 1) the association between everyday discrimination and generalized anxiety disorder (GAD) and 2) whether church-based relationships buffer the negative effects of everyday discrimination on GAD among older African Americans. Logistic regression analyses were conducted using data from 670 African American respondents age 55 and older from the National Survey of American Life: Coping with Stress in the 21st Century. More frequent experiences of everyday discrimination was associated with higher odds of meeting criteria for GAD. Significant interactions indicated that frequent contact with church members and high levels of subjective closeness to church members buffered against the negative effects of discrimination on GAD. Interventions that focus on the use of church members for support capitalize on a major strength among older African Americans.

